# Circulating Soluble CD163: A Potential Predictor for the Functional Outcome of Acute Ischemic Stroke

**DOI:** 10.3389/fneur.2021.740420

**Published:** 2021-12-14

**Authors:** Houchao Sun, Xiaogang Zhang, Jingxi Ma, Zhao Liu, Yunwen Qi, Li Fang, Yongling Zheng, Zhiyou Cai

**Affiliations:** ^1^Department of Neurology, Chongqing Medical University, Chongqing, China; ^2^Department of Neurology, Chongqing General Hospital, University of Chinese Academy of Sciences, Chongqing, China; ^3^Chongqing Key Laboratory of Neurodegenerative Diseases, Chongqing, China; ^4^Chongqing Key Laboratory of Neurology, Department of Neurology, First Affiliated Hospital of Chongqing Medical University, Chongqing, China

**Keywords:** acute ischemic stroke, biomarker, soluble CD163, short-term, functional outcome

## Abstract

**Background:** CD163 is a transmembrane glycoprotein receptor expressed on innate immune cells that sheds from the cell membrane and circulates as a soluble form (sCD163). This study aimed to investigate the circulating levels and clinical relevance of soluble CD163 (sCD163) in acute ischemic stroke (AIS).

**Methods:** This study recruited 300 patients with AIS and 78 healthy controls. The patients were followed up for 1 month to observe the functional outcomes. The neurological functions of the patients were assessed using the NIH Stroke Scale (NIHSS) and the modified Rankin Scale (mRS). The plasma concentrations of sCD163 at the baseline (patient admission) were determined by ELISA.

**Results:** We found that patients with AIS had significantly higher plasma sCD163 concentrations than the healthy control. Patients with high sCD163 concentrations had better functional outcomes than patients with low sCD163 concentrations. The plasma sCD163 concentrations were positively associated with the NIHSS scores and infarction volume at the baseline. The plasma sCD163 was positively associated with the improvement of the NIHSS scores but was negatively associated with the risk of poor functional outcomes during follow-up.

**Conclusions:** These findings indicate that circulating sCD163 is a potential biomarker that is associated with disease severity and the functional outcome of AIS.

## Introduction

Stroke is one of the leading causes of death and disability worldwide (1). Biomarkers with the potential in identifying patients with a risk of having poor clinical outcomes are critical for aggressive monitoring and therapeutic interventions in these subjects. A panel of blood-based biomarkers is suggested to be predictive for the severity and prognosis of acute ischemic stroke (AIS) (2, 3).

The plasma membrane glycoprotein receptor CD163 is a member of the scavenger receptor cysteine-rich (SRCR) superfamily class B that is mostly expressed on monocytes and macrophages. CD163 could shed from cell membranes to release soluble CD163 (sCD163) upon stimulation by inflammatory stimuli (4). sCD163 has been suggested to be biomarkers of many diseases, such as infectious diseases (5), tumors (6), and autoimmune diseases (7). sCD163 is increased in patients with intracranial hemorrhage and is associated with the improvement of neurological functions by promoting hematoma absorption (8). AIS involves local immune responses that encompass brain resident microglia and monocytes infiltrating from the circulation (9). The CD163 induced anti-inflammatory effects of monocytic cells are suggested to be involved in the pathogenesis of AIS (10). Animal studies have demonstrated that CD163 is upregulated following AIS (11). However, the changes of circulating sCD163 in patients with AIS are unknown. Therefore, this study aims to investigate the levels and clinical relevance of sCD163 in patients with AIS.

## Materials and Methods

### Subjects

Patients with their first AIS (to exclude the effects of previous AIS events on circulating sCD163 concentrations) who visited the Department of Neurology, Chongqing General Hospital, University of Chinese Academy of Sciences during January 1, 2019, and January 31, 2020, were screened. The inclusion criteria included: (1) patients with newly onset AIS; (2) who visited the hospital within 24 h after symptom onset; (3) who are willing to participate. Seventy-eight age and sex-matched healthy subjects were recruited as controls from the healthy examination center of the same hospital. Subjects with diseases that might influence the circulating sCD163 levels were excluded from participation. Therefore, subjects were excluded if they have: (1) co-existing infections; (2) any types of tumors; (3) any types of autoimmune diseases; (4) other conditions that may influence the blood sCD163 levels, such as cirrhosis and severe inflammatory diseases; (5) declined to participate in this study. The patients were screened for eligibility for participation right after admission. However, although diabetes mellitus may contribute to the alteration of sCD163 (12), patients with diabetes mellitus were not excluded as it is a significant risk factor of AIS. Finally, 300 patients and 78 healthy controls were recruited in this study after excluding the subjects who failed for inclusion. Written informed consents for participation in this study and blood sampling were obtained from the subjects or their legal relatives. This study conformed with the principles of the Declaration of Helsinki and was approved by the investigational review board of the Chongqing General Hospital, University of Chinese Academy of Sciences.

### Clinical Evaluation

At baseline (time of patient admission), the demographic information such as age, sex, and body mass index (BMI), pre-stroke medical history including oral antiplatelet or anticoagulants drug use, comorbidities including hypertension, diabetes mellitus, hypercholesteremia, and atrial fibrillation were collected and assessed right after admission. AIS was diagnosed according to the WHO Multinational Monitoring of Trends and Determinants in Cardiovascular Disease (WHO-MONICA) criteria with verification by MRI performed within 12 h after admission. The neurological deficits were examined with the National Institutes of Health Stroke Scale (NIHSS) upon admission (13), performed by a certified stroke neurologist. The AIS subtype was determined with the Trial of Org 10172 in Acute Stroke Treatment (TOAST) criteria (14).

### Patients' Follow-Up

The patients were followed up for 1 month since admission for the observation of functional outcomes. The primary endpoint was neurological functions 1 month since admission. The functional outcomes were assessed using NIHSS and the modified Rankin Scale (mRS) (15), which were blinded to the plasma sCD163 concentrations.

### Blood Sampling and Measurement of CD163

Blood sampling was conducted right after patient admission before they receive any medical interventions. The blood was centrifuged right after collection at 20°C, spun at 2,000 g for 10 min, and stored at −80°C for biochemical analysis. The plasma sCD163 levels were determined using a human CD163 ELISA kit (Abcam, USA) according to the manufactural instructions. To preserve the linearity of the assays, samples containing high concentrations of sCD163 were diluted with an appropriate amount of calibrator diluent. The minimum detectable dose of sCD163 was 1.377 ng/ml, which was significantly lower than the sCD163 concentrations of the subjects in this study. Each test was conducted in duplicates and the means were used for statistical analysis.

### Statistical Analysis

If continuous variables were normally distributed, an independent *t*-test was used, and if not, a Mann-Whitney *U*-test was used. Two-sample tests of proportions were used to compare the proportions for the categorical variables. The comparisons of means among groups were conducted using one-way ANOVA and the comparisons of the rate of NIHSS change during the follow-up between groups were conducted using two-way ANOVA. Spearman correlation analyses were conducted to assess the association between plasma sCD163 concentrations and NIHSS or infarction volume. A linear regression model was utilized to investigate the association between the plasma sCD163 levels and functional improvement (as indicated by the change of NIHSS during follow-up). A logistical regression model was utilized to evaluate the risk factors of poor functional outcome (as indicated by mRS≥4) during follow-up. We first fitted univariate models with a single candidate variable at one time. The potential risk factors as determined by *p* < 0.05 were included in the final multivariate regression model. Statistical analyses were conducted using SPSS statistical package version 24 (IBM SPSS Statistics for Windows, Armonk, NY, USA).

## Results

### Demographic Characteristics of Subjects

**Figure 1:**
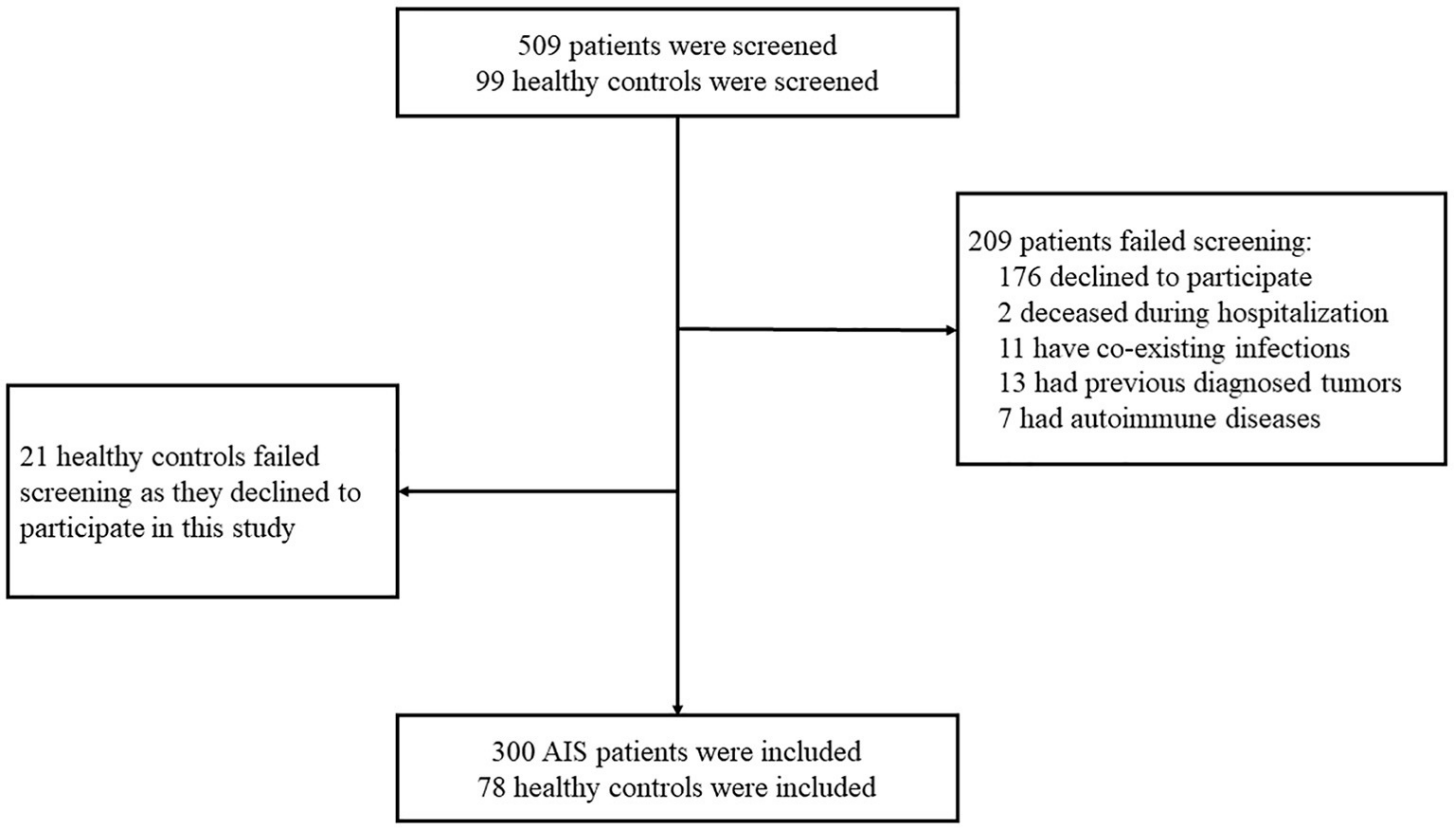
Subjects screening flowchart.

Three-hundred-nine patients with AIS and 99 healthy controls were screened for eligibility for participation in this study. Two-hundred-nine patients failed screening due to the following reasons: 176 patients declined to participate, 2 patients deceased during hospitalization, 11 patients had co-existing infections, 13 patients had previously diagnosed tumors, and 7 patients had autoimmune diseases. Twenty-one healthy controls declined to participate in this study. Finally, 300 AIS patients and 78 healthy controls participated in this study (Figure 1).

The patients were further divided into the high and low plasma sCD163 groups, respectively, according to their plasma sCD163 concentrations. The patients had significantly higher median BMI than the healthy controls. No significant differences in the mean age, percentages of males, frequencies of subjects with a history of smoking, antiplatelet drug use, antithrombotic drug use, a family history of stroke, hypertension, diabetes mellitus, hypercholesteremia, and atrial fibrillation between the patients and controls were observed. The high sCD163 group had a significantly higher proportion of males, a higher median diffusion-weighted imaging (DWI) hyperintensity volume, higher NIHSS scores at baseline, and higher frequencies of lacunar-type stroke than the low sCD163 group. However, there was no difference in the mean age, median BMI, frequency of smoking, antiplatelet drug use, antithrombotic drug use, family history of stroke, comorbidities including hypertension, diabetes mellitus, hypercholesteremia, and atrial fibrillation between the two groups. Besides, the median DWI hyperintensity volume, the median NIHSS and mRS scores at follow-up, frequencies of atherothrombotic, cardioembolic, and unknown type stroke, frequencies of hemorrhagic transformation, and recurrent AIS during follow-up were not significantly different between the high and low sCD163 groups (Table 1).

**Table 1:** Demographic data of subjects.

**Variables**	**Control**	**Low sCD163 group**	**High sCD163 group**	***P*-value Control**	***P*-value Low**
	**(*n* =78)**	**(*n* = 150)**	**(*n* = 150)**	**vs. patients**	**vs. High**
Age, Mean (SD)	62.86 (10.89)	64.83 (9.20)	65.16 (9.34)	0.082	0.756^a^
Male, No. (%)	48 (61.54)	95 (63.33)	75 (50.00)	0.520	**0.027^b^**
BMI, Median (IQR)	23.85 (22.28, 25.33)	24.68 (23.14, 25.76)	24.19 (22.96, 25.37)	**0.029**	0.086^c^
Smoking history, No. (%)	7 (8.97)	11 (7.33)	16 (10.67)	0.437	0.420^b^
Antiplatelet drug use, No. (%)	9 (11.54)	21 (14.00)	19 (12.67)	0.850	0.865^b^
Antithrombotic drug use, No. (%)	4 (5.13)	13 (8.67)	6 (4.00)	1.000	0.153^b^
Family history of stroke, No. (%)	3 (3.85)	9 (6.00)	9 (6.00)	0.587	1.000^b^
**Comorbidities**
Hypertension, No. (%)	21 (26.92)	52 (34.67)	51 (34.00)	0.226	1.000^b^
Diabetes Mellitus, No. (%)	13 (16.67)	26 (17.33)	23 (15.33)	1.000	0.755^b^
Hypercholesteremia, No. (%)	6 (7.69)	9 (6.00)	19 (12.67)	0.825	0.072^b^
Arial fibrillation, No. (%)	7 (8.97)	13 (8.67)	6 (4.00)	0.451	0.153^b^
DWI hyperintensity volume, ml (SD)	NA	24.56 (8.86)	32.36 (7.53)	NA	**0.000** ^**a**^
NIHSS at baseline, Median (IQR)	NA	8 (3, 13)	15 (11.75, 19)	NA	**0.000^c^**
NIHSS at follow-up, Median (IQR)	NA	4 (1, 9)	6 (3, 7)	NA	0.595^c^
mRS at follow-up, Median (IQR)					
**Stroke etiology**
Atherothrombotic, No. (%)	NA	122 (81.33)	131 (87.33)	NA	0.204^b^
Cardioembolic, No. (%)	NA	13 (8.67)	6 (4.00)	NA	0.153^b^
Lacunar, No. (%)	NA	13 (8.67)	4 (2.67)	NA	**0.043^b^**
Unknown, No. (%)	NA	2 (1.33)	9 (6.00)	NA	0.061^b^
**Complication**
Hemorrhagic transformation, No. (%)	NA	3 (2.00)	7 (4.67)	NA	0.335^b^
Recurrent AIS, No. (%)	NA	4 (2.67)	1 (0.67)	NA	0.371^b^
rtPA treatment, No. (%)	NA	13 (8.67)	16 (10.67)	NA	0.697^b^
mRS scores, Median (IQR)	NA	2 (0, 4)	3 (2, 3)	NA	0.641^c^

*IQR, Inter-Quartile Range; BMI, Body Mass Index; TICS, Telephone Interview of Cognitive Status 40; NIHSS, National Institutes of Health Stroke Scale; NA, not applicable*.

a *Unpaired t-test;*

b *Pearson χ^2^-test;*

c *Mann-Whitney U-test. Bold values represents statistically significant*.

### Plasma Concentrations and Clinical Relevance of sCD163 in AIS

**Figure 2:**
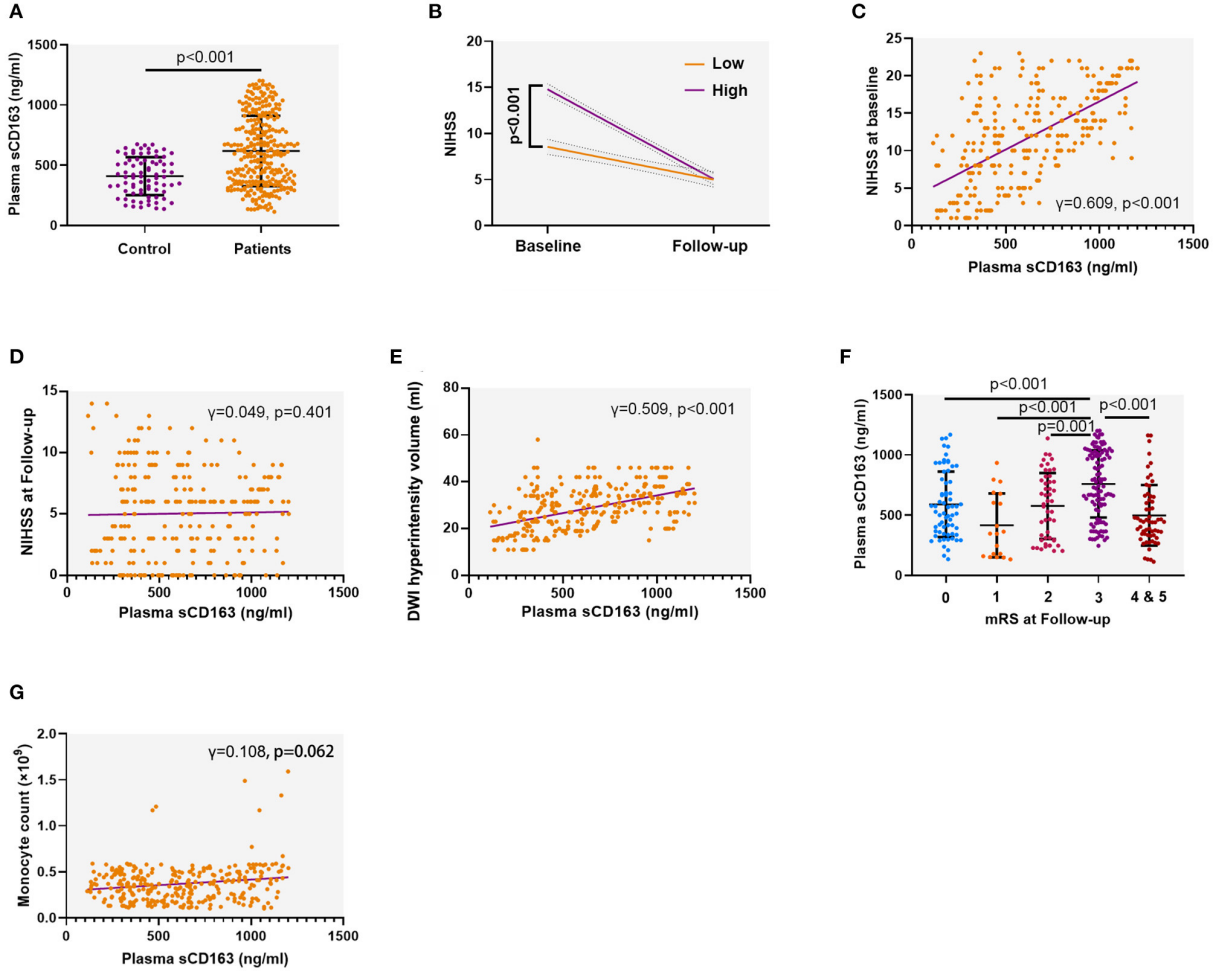
Plasma soluble CD163 (sCD163) is increased in patients with acute ischemic stroke (AIS) and associated with disease severity. **(A)** Plasma sCD163 concentrations in AIS patients and control. Independent sample *t*-test. **(B)** The trajectory of neurological function as indicated by the change of the National Institutes of Health Stroke Scale (NIHSS) during follow-up. Two-way ANOVA. **(C)** Association between plasma sCD163 concentrations and NIHSS at baseline. Spearman correlation analysis. **(D)** Association between plasma sCD163 concentrations and NIHSS at follow-up. Spearman correlation analysis. **(E,F)** Association between plasma sCD163 concentrations and diffusion-weighted imaging (DWI) hyperintensity volume. **(G)** Association between plasma sCD163 concentrations and circulating monocyte count. DWI, Diffusion-weighted imaging. Spearman correlation analysis.

We first compared the sCD163 concentrations between the patients with AIS and the control. We found that patients with AIS had significantly higher plasma sCD163 concentrations than the control (mean ± SD: 618.1 ± 292.0 ng/ml vs. 408.8 ± 157.5 ng/ml, *p* < 0.001) (Figure 2A). The high sCD163 group had better functional improvement as indicated by the change of NIHSS during follow-up than the low sCD163 group (*p* < 0.001) (Figure 2B). The plasma sCD163 concentrations were positively associated with the NIHSS at baseline (γ = 0.609, *p* < 0.001) and the DWI hyperintensity volume (γ = 0.509, *p* < 0.001) (Figures 2C,D). However, plasma sCD163 concentrations were not significantly associated with NIHSS at follow-up (γ = 0.049, *p* = 0.401) (Figure 2E). Patients with an mRS = 3 had significantly higher sCD163 concentrations than patients with an mRS = 1 (*p* < 0.001), 2 (*p* < 0.001), 4 (*p* < 0.001), and 5 (*p* < 0.001) (Figure 2F). Plasma sCD163 concentrations were positively associated with peripheral monocyte count, but no significance had been achieved (γ = 0.108, *p* = 0.062) (Figure 2G).

### Factors Associated With Plasma sCD163 Levels

We utilized a linear regression model to investigate potential factors that were associated with plasma sCD163 levels. We found that hypercholesteremia and DWI hyperintensity volume were significantly associated with plasma sCD163 levels. These two factors along with diabetes mellitus, which is well-validated to be associated with sCD163 levels, were excluded from subsequent analysis (Table 2).

**Table 2:** A linear regression model to investigate potential factors associated with plasma sCD163 levels.

**Variables**	**β unadjusted**	**S.E**.	**β adjusted**	***P*-value**
Constant	264.512	283.288		0.351
Age	−0.241	1.611	−0.008	0.881
Male	−56.423	33.021	−0.096	0.089
BMI	−2.757	10.833	−0.014	0.799
Hypertension	5.681	32.151	0.009	0.860
Diabetes mellitus	−66.343	40.596	−0.084	0.103
Hypercholesteremia	**116.374**	**52.105**	**0.116**	**0.026**
Smoking history	19.139	58.664	0.019	0.744
Family history of stroke	37.071	70.546	0.030	0.600
Stroke type	19.856	21.462	0.050	0.356
DWI hyperintensity Volume	**15.328**	**1.709**	**0.477**	**0.000**
Antiplatelet drug use	43.171	44.491	0.050	0.333
Antithrombotic drug use	−81.982	63.849	−0.068	0.200

*Dependent variable: sCD163 concentrations. Bold values represents statistically significant*.

### Association Between Plasma sCD163 Levels and Functional Improvement During Follow-Up

We first utilized a linear regression model to investigate the association between plasma sCD163 levels and functional improvement during follow-up as indicated by the decrease of NIHSS. Factors that are potentially associated with sCD163 levels were excluded from the model, including hypercholesteremia, DWI hyperintensity volume, and diabetes mellitus. Male sex and higher sCD163 concentrations were found to be positively associated with functional improvement during follow-up (Table 3).

**Table 3:** A linear regression model to evaluate the association between plasma sCD163 levels and functional improvement during follow–up.

**Variables**	****β** unadjusted**	**S.E**.	****β** adjusted**	***P*-value**
Constant	2.177	3.616		0.548
Age	−0.015	0.021	−0.029	0.457
Male	**−1.885**	**0.430**	**−0.186**	**0.000**
BMI	−0.039	0.141	−0.012	0.784
Hypertension	0.152	0.417	0.014	0.717
Smoking history	−0.654	0.758	−0.037	0.389
Family history of stroke	.001	0.917	0.000	0.999
Stroke type	0.018	0.274	0.003	0.947
Antiplatelet drug use	0.491	0.571	0.033	0.391
Antithrombotic drug use	−0.432	0.829	−0.021	0.603
sCD163 concentrations	**0.012**	**0.001**	**0.698**	**0.000**

*Dependent variable: Functional improvement as indicated by the change of NIHSS during follow–up (NIHSS at baseline—NIHSS at follow–up). Factors that are potentially associated with sCD163 levels were excluded from the model. Bold values represents statistically significant*.

### Association Between Plasma sCD163 Concentrations and Poor Functional Outcome During Follow-Up

We next utilized a logistical regression model to investigate the association between plasma sCD163 concentrations and poor functional outcome during follow-up as indicated by mRS ≥ 4. In the univariate analyses, stroke type was found to be associated with poor functional outcomes during follow-up. Higher sCD163 concentrations were found to be protective factors of poor functional outcome during follow-up, and these associations remained significant in the multivariate analyses (Table 4).

**Table 4:** A logistic regression model to evaluate risk factors of poor functional outcome during follow-up.

**Variables**	**Univariable ORs (95%CI)**	***P* value**	**Multivariable ORs (95%CI)**	***P*-value**
Age	0.987 (0.957, 1.018)	0.394		
Sex, male vs. female	1.413 (0.789, 2.533)	0.245		
BMI	1.044 (0.865, 1.260)	0.656		
Hypertension, yes vs. no	0.782 (0.424, 1.442)	0.430		
Diabetes mellitus, yes vs. no	0.509 (0.206, 1.259)	0.144		
Hypercholesteremia, yes vs. no	0.858 (0.312, 2.358)	0.766		
Smoking history, yes vs. no	0.737 (0.208, 2.616)	0.637		
Family history of stroke, yes vs. no	1.159 (0.446, 3.011)	0.762		
Stroke type	0.456 (0.225, 0.925)	**0.030**	**0.392 (0.184, 0.835)**	**0.015**
Antiplatelet drug use, yes vs. no	0.532 (0.199, 1.423)	0.209		
Antithrombotic drug use, yes vs. no	0.737 (0.208, 2.616)	0.637		
sCD163 concentrations, high vs. low	0.351 (0.191, 0.644)	**0.001**	**0.998 (0.997, 0.999)**	**0.000**

*Dependent variable: Poor outcome as indicated by mRS = 4 or 5 at follow-up. Factors that are potentially associated with sCD163 levels were excluded from the model. Bold values represents statistically significant*.

## Discussion

In this study, we investigated the levels and clinical relevance of sCD163 in AIS. We found that patients with AIS had increased circulating sCD163 concentrations. sCD163 concentrations were associated with the severity and prognosis of AIS. Specifically, sCD163 was positively associated with the improvement of neurological functions but negatively associated with the risk of poor prognosis in AIS during follow-up.

Previous studies suggest that the AIS-associated inflammatory component is partly driven by the myeloid immune compartment, including microglia, peripheral monocytes, and macrophages (16). Numerous studies identified alterations in immune biomarkers in the cerebral spinal fluid (CSF) and blood that are associated with the severity and prognosis of AIS (17). However, few of these biomarkers are cell type-specific and thus provide limited information on cellular relevance in the pathogenesis of AIS. Here, we show that plasma sCD163, a monocyte/macrophage-specific biomarker, was increased in AIS, hence suggesting increasing monocytic activation after AIS. sCD163 was associated with the disease severity as reflected by the NIHSS and infarction volume at the baseline. In previous studies, sCD163 is suggested to be increased in a panel of neurological diseases, such as intracranial hemorrhage (18), Parkinson's disease (19), Alzheimer's disease (20), and multiple sclerosis (21). These diseases without exception involve monocytic activation. Therefore, we suppose that the increase in sCD163 levels after AIS is indicative of monocytic activation, which is a well-documented phenomenon in AIS (22). This notion is further supported by our findings that plasma sCD163 concentrations were associated with peripheral monocyte count, although no significance had been achieved. Monocyte activation may attenuate with the diminishment of the post-AIS inflammation and the recovery of the disease (10), which is supported by the loss of association between plasma sCD163 concentrations and NIHSS at follow-up when neurological functions had been significantly improved.

Ischemic stroke causes local inflammation, which involves both the activation of resident microglia and infiltrating of peripheral immune cells, including monocytes. Blocking monocyte recruitment post-AIS abolishes long-term neurological recovery and decreases the tissue expression of anti-inflammatory factors including CD163 (10). In a previous study, post-mortem brain specimens from patients with AIS showed the time-dependent accumulation of CD163+ monocytes in the ischemic parenchyma (23), indicating that the increase of CD163 expression post-AIS might be a physical protective mechanism against ischemia-associated neuronal damage. In accordance with this speculation, we found that patients with high baseline sCD163 levels have better improvement of neurological functions than those with low baseline sCD163 levels. Furthermore, we found in the regression analyses that sCD163 levels were positively associated with the improvement of neurological functions but negatively associated with the risk of poor clinical outcomes, further supporting the hypothesis that monocyte activation may serve as a protective mechanism of neuronal injury repairment post-AIS.

There are several limitations of this study. First, there is a phenomenon in this study that could not be reasonably explained that sCD163 concentrations were highest in patients with an mRS score = 3 but were relatively low in patients with an mRS score = 4 and 5. This inconsistency may limit the confidence of the present study, thus, further investigations with larger sample sizes are needed to address a more solid conclusion. Second, the novelty of this study is limited by previous findings that sCD163 levels are increased after AIS onset. Actually, previous studies demonstrate that CD163 in the brain showed a dynamic change with a peak level at the 3rd day after AIS in animal models (10). Furthermore, we did not investigate the dynamic change of sCD163 post-AIS, which is of importance to interpret the role of CD163 in the pathogenesis of AIS from a clinical perspective. Therefore, further studies are needed to investigate the change of sCD163 in different stages of AIS. Moreover, the exact time of the blood draw for sCD163 levels was not consistent as far as time from stroke symptom onset in patients in the study. Most importantly, the data presented is correlative and only demonstrates a potential relationship between functional outcomes following AIS and increased plasma levels of CD163, which might be a true phenomenon but also unrelated to disease severity and functional outcomes. This study lacks experimental evidence to support the interpretations.

## Data Availability Statement

The raw data supporting the conclusions of this article will be made available by the authors, without undue reservation.

## Ethics Statement

This study was approved by the investigational review board of the Chongqing General Hospital, University of Chinese Academy of Sciences. The patients/participants provided their written informed consent to participate in this study.

## Author Contributions

ZC and HS designed the study and drafted the manuscript. HS, XZ, JM, and ZL collected the samples and analyzed the data. YQ and LF supervised the project. YZ and LF were responsible for the clinical assessment of subjects. All authors contributed to the article and approved the submitted version.

## Funding

The work was supported by the Science and Technology Committee of Yuzhong District of Chongqing (20180142), Natural Science Foundation of Chongqing (cstc2020jcyj-msxmX0058), Chongqing General Hospital (2019ZDXM03), and Chongqing Municipal Health Commission (2020MSXM106).

## Conflict of Interest

The authors declare that the research was conducted in the absence of any commercial or financial relationships that could be construed as a potential conflict of interest.

## Publisher's Note

All claims expressed in this article are solely those of the authors and do not necessarily represent those of their affiliated organizations, or those of the publisher, the editors and the reviewers. Any product that may be evaluated in this article, or claim that may be made by its manufacturer, is not guaranteed or endorsed by the publisher.
